# Association between S100 calcium-binding protein A12 and sepsis associated-acute kidney injury: a prospective cohort study

**DOI:** 10.1186/s12882-026-04760-0

**Published:** 2026-01-22

**Authors:** Huanqin Liu, Mengjia Yu, Yumei Mao, Qingjie Xue, Yanan Lv, Feng Qu, Jikui Shi

**Affiliations:** 1 Department of Critical Care Medicine, Jining No. 1 People’s Hospital, Jining, Shandong 272000 China; 2 Department of Intensive Care Medicine, Affiliated Hospital of Jining Medical University, Jining, Shandong 272029 China; 3 Department of Critical Care Medicine, East Sichuan Hospital of Sichuan Provincial People’s Hospital (Dazhou First People’s Hospital), Dazhou, Sichuan 635002 China; 4 School of Basic Medicine, Jining Medical University, Jining, Shandong 272067 China; 5 School of Clinical Medicine, Jining Medical University, Jining, Shandong 272067 China

**Keywords:** S100 calcium-binding protein A12, Sepsis-associated acute kidney injury, Sepsis, Biomarker, Cohort study, Logistic regression

## Abstract

**Background:**

Sepsis-associated acute kidney injury (SA-AKI) is a common and severe complication in critically ill patients, yet early diagnostic biomarkers remain limited. S100A12, a pro-inflammatory calcium-binding protein, may contribute to sepsis-induced renal injury through oxidative and immune-mediated mechanisms. This study aimed to investigate the association between plasma S100A12 levels and the risk of SA-AKI in a prospective sepsis cohort.

**Methods:**

In this prospective cohort study, 202 adult patients with sepsis were consecutively enrolled. Plasma S100A12 levels were measured at enrollment. Associations between S100A12 (continuous and tertiles) and SA-AKI were assessed using multivariable logistic regression (Models 1–3) with progressive adjustment for clinical and laboratory covariates. Restricted cubic spline (RCS) regression and Cox proportional-hazards models were applied to evaluate dose–response and temporal relationships. Predictive performance was assessed using receiver-operating characteristic (ROC) analysis.

**Results:**

SA-AKI occurred in 59.9% of participants. S100A12 concentrations were positively associated with SA-AKI risk. After adjustment for confounders, elevated S100A12 was independently associated with SA-AKI (odds ratio 1.17, 95% CI 1.11–1.24, *p* < 0.001). No significant interactions were observed across predefined subgroups (all p for interaction > 0.05). The area under the ROC curve (AUC) was 0.839 (95% CI 0.783–0.895).

**Conclusions:**

Higher plasma S100A12 levels were independently associated with the development of sepsis-associated acute kidney injury in critically ill patients with sepsis. S100A12 may represent a candidate biomarker for early risk stratification in SA-AKI; however, external validation in larger, multicenter cohorts is required before any potential clinical application.

**Trial registration:**

This prospective observational study was retrospectively registered in the Chinese Clinical Trial Registry (ChiCTR2400094790) on December 27, 2024, after ethics approval, as no interventional procedures were performed.

**Supplementary Information:**

The online version contains supplementary material available at 10.1186/s12882-026-04760-0.

## Introduction

Sepsis is a life-threatening syndrome characterized by a dysregulated host response to infection, leading to organ dysfunction [1]. It remains a major global cause of morbidity and mortality, with its incidence continuing to rise [2–5]. Among the various organ failures associated with sepsis, acute kidney injury (AKI) is one of the most common and devastating. Sepsis-associated AKI (SA-AKI) contributes substantially to poor outcomes, including increased mortality, cardiovascular complications, and progression to chronic kidney disease (CKD) [6]. According to the Kidney Disease: Improving Global Outcomes (KDIGO) criteria and Sepsis-3 definitions, SA-AKI is defined as new-onset AKI within 7 days of sepsis diagnosis [7]. Early identification of patients at risk for AKI or those likely to develop severe or persistent AKI is crucial for timely intervention to prevent further renal injury [8, 9].

S100 calcium-binding protein A12 (S100A12) is a damage-associated molecular pattern (DAMP) predominantly released by activated neutrophils and monocytes. It interacts with the receptor for advanced glycation end-products (RAGE) and toll-like receptor (TLR) 4, amplifying inflammatory signaling and promoting cytokine release [10–12]. Previous studies have demonstrated significant alterations in S100A12 levels among patients with sepsis, highlighting its role in infection, inflammation, and immune regulation [13–16]. Notably, elevated postoperative S100A12 levels have been associated with early AKI after cardiac surgery [17]. These findings suggest that S100A12 may serve as a link between systemic inflammation and renal dysfunction.

However, despite these observations, the relationship between S100A12 and SA-AKI remains incompletely understood. A recent study found no significant difference in S100A12 levels between septic patients with and without AKI [18], underscoring conflicting evidence and the need for further research. While S100A12 is mechanistically linked to neutrophil activation, endothelial injury, and amplification of the inflammatory cascade—pathways central to sepsis-induced renal damage [19, 20]—its temporal behavior and prognostic relevance in SA-AKI have not been systematically evaluated. Most prior studies were cross-sectional or limited in sample size, precluding assessment of dynamic changes and predictive performance. A prospective cohort study integrating serial biomarker measurements with clinical outcomes is therefore warranted to clarify the role of S100A12 as a mechanistically relevant and potentially predictive biomarker of SA-AKI.

Accordingly, this study aimed to prospectively evaluate the association between plasma S100A12 levels and the development of SA-AKI in septic patients. We further sought to assess its predictive value for early risk stratification and explore the potential pathophysiological implications of S100A12 in SA-AKI. To our knowledge, this is the first prospective study to systematically examine S100A12 in SA-AKI, providing new insights into its clinical utility and mechanistic relevance.

## Methods

### Study design and data sources

This prospective cohort study was conducted in the Department of Critical Care Medicine at Jining No. 1 People’s Hospital (Shandong, China) between September 2023 and June 2025. The study adhered to the principles of the Declaration of Helsinki and was approved by the hospital’s Ethics Committee (Approval No. KYLL-202308-137). Written informed consent was obtained from all participants or their legally authorized representatives before enrollment. The study followed the Strengthening the Reporting of Observational Studies in Epidemiology (STROBE) guidelines [21].

All adult patients (≥18 years) admitted to the intensive care unit (ICU) for the first time during the study period were screened for eligibility. Exclusion criteria included an ICU stay of < 24 hours, active malignancy, pre-existing chronic kidney disease, absence of sepsis diagnosis, or missing plasma S100A12 measurements. Patients were stratified into tertiles according to baseline S100A12 levels for subsequent analyses. In addition, healthy volunteers (≥18 years, without recent infection or chronic disease) were recruited as controls.

### Data collection and definitions

Clinical data were prospectively extracted from the hospital’s electronic medical record system. Plasma S100A12 concentrations obtained within 24 hours of ICU admission were measured using a commercially available human S100A12 enzyme-linked immunosorbent assay (ELISA) kit (Meimian Biotechnology Co., Ltd., Jiangsu, China; Cat. No. MM-12405H1). According to the manufacturer’s protocol, plasma samples were diluted five-fold before analysis. Standard solutions (S0–S5) were prepared at concentrations of 0, 3, 6, 12, 24, and 48 ng/mL. Absorbance was measured at 450 nm, and sample concentrations were calculated from the standard curve. All samples were assayed in duplicate; the mean value was used for analysis. The intra- and inter-assay coefficients of variation were < 8% and < 10%, respectively. Hemolyzed or lipemic samples were excluded, and aliquots were stored at −80 °C until testing.

Demographic characteristics, including age, sex, and body mass index (BMI), were recorded at intensive care unit (ICU) admission. Comorbidities comprised hypertension (HBP), diabetes mellitus (DM), and cardiovascular disease (CVD). Disease severity was assessed using the Sequential Organ Failure Assessment (SOFA) and Acute Physiology and Chronic Health Evaluation II (APACHE II) scores. Laboratory parameters measured at enrollment included C-reactive protein (CRP), neutrophil count, procalcitonin (PCT), serum creatinine (Cr), coagulation disorder status, N-terminal pro-brain natriuretic peptide (NT-proBNP), arterial oxygen pressure (PaO₂), blood lactate, and mean arterial pressure (MAP). Clinical variables comprised the primary site of infection—classified as pulmonary infection or abdominal infection—the 24-h urine output, and 7-day cumulative fluid balance. Major ICU interventions were documented, including continuous renal replacement therapy (CRRT), mechanical ventilation (MV), vasopressor therapy, and exposure to highly nephrotoxic antibiotics before AKI onset. Complications such as acute respiratory distress syndrome (ARDS) and septic shock were defined according to standard criteria.

Clinical outcomes included hospital length of stay, ICU length of stay, 28-day mortality, and overall survival time.

Sepsis was diagnosed in accordance with the Sepsis-3 definition [1], i.e., life-threatening organ dysfunction caused by a dysregulated host response to infection, defined as an increase of ≥ 2 points in the SOFA score. Infections and comorbidities were identified using International Classification of Diseases, Ninth and Tenth Revisions (ICD-9/10) codes.

### Outcomes

The primary outcome was the occurrence of SA-AKI within seven days of ICU admission [6]. AKI was defined according to the Kidney Disease: KDIGO criteria [22] as an acute increase in serum Cr ≥ 0.3 mg/dL within 48 hours or urine output < 0.5 mL/kg/h for ≥ 6 hours. When the interval between two Cr measurements was < 6 hours, urine output criteria were applied. AKI was considered present if patients met criteria for KDIGO stage 1 or higher. Patients who died within 7 days without meeting AKI criteria were treated as not having the event in the primary 7-day binary outcome analysis; in time-to-event analyses, they were censored at the time of death.

### Statistical analyses

#### Software and descriptive statistics

All statistical analyses were performed using R software (version 4.3.3; R Foundation for Statistical Computing, Vienna, Austria; http://www.R-project.org) and the Free Statistics software (version 2.3). Continuous variables were expressed as mean ± standard deviation (SD) or median (interquartile range [IQR]) and compared using one-way ANOVA or the Kruskal–Wallis test as appropriate. Categorical variables were presented as counts (%) and compared using the χ^2^ test or Fisher’s exact test. A two-sided *p* value < 0.05 was considered statistically significant.

#### Association analyses between S100A12 and SA-AKI

Patients were categorized into tertiles according to plasma S100A12 levels at ICU admission: tertile 1 (T1: ≤38.46 ng/mL), tertile 2 (T2: 38.47–52.20 ng/mL), and tertile 3 (T3: > 52.20 ng/mL). Associations between S100A12 (as a continuous variable and by tertiles) and the risk of SA-AKI were evaluated using multivariable logistic regression. Covariates were selected a priori based on clinical relevance and prior literature, with univariate analyses used to inform, but not determine, model specification. Prior to multivariable model construction, multicollinearity among explanatory variables was assessed using variance inflation factors (VIFs) and pairwise correlation analyses. No evidence of problematic multicollinearity was observed.

Model 1: adjusted for age, gender, BMI, and HBP.

Model 2: adjusted for Model 1 plus SOFA score, APACHE II score, CRP, neutrophil count, PCT, coagulation disorder, NT-proBNP, PaO₂, blood lactate, and blood glucose.

Model 3: adjusted for Model 2 plus MV, vasopressor therapy, MAP, septic shock, highly nephrotoxic antibiotic exposure before AKI (HAAKI), and 7-day cumulative fluid balance.

Sensitivity model (Model 4): An independently specified multivariable logistic regression model constructed as a sensitivity analysis, adjusting for age, gender, BMI, HBP, SOFA score, CRP, lactate, MAP, HAAKI, serum albumin, serum Cr, and 7-day cumulative fluid balance.

#### Time-to-event analysis

To examine the temporal relationship between plasma S100A12 levels and the development of SA-AKI, a time-to-event analysis was conducted using the Cox proportional hazards model.

The time variable was defined as the duration (in days) from ICU admission to the first occurrence of SA-AKI within 7 days. Patients who did not develop SA-AKI during this period were censored at the time of ICU discharge or at day 7, whichever came first.

Hazard ratios (HRs) and 95% CIs were estimated using the same covariate adjustment strategy as in the logistic regression analysis (Model 1–3).

In addition to the regression analyses, a time-to-event analysis was conducted to visualize the temporal distribution of SA-AKI occurrence across S100A12 tertiles. Kaplan–Meier curves were generated, and group differences were compared using the log-rank test.

#### Nonlinearity and trend analyses

To assess linearity, restricted cubic spline (RCS) regression was applied with S100A12 as a continuous variable. Trend analyses across tertiles were performed treating tertiles as ordinal variables, with p for trend adjusted for Model 3 covariates.

#### Subgroup and interaction analyses

Subgroup and interaction analyses were conducted according to age, gender, body mass index (BMI), SOFA score, APACHE II score, highly nephrotoxic antibiotic exposure before AKI (HAAKI), and infection site (abdominal vs. non-abdominal).

#### Discrimination and predictive performance

The discriminative ability of plasma S100A12 for SA-AKI was assessed using receiver operating characteristic (ROC) curve analysis. The area under the curve (AUC) with 95% confidence interval (CI) quantified predictive performance. The optimal cutoff was determined using the Youden index, and corresponding sensitivity, specificity, and overall accuracy were reported.

#### Missing data handling

Missing data ranged from 0.5% to 10.4%. Mean, median, and mode single-imputation methods were applied for normally distributed, non-normal continuous, and categorical variables, respectively. Because the proportion of missingness was low, analyses using complete-case and imputed datasets produced consistent results.

#### Robustness analyses

##### E-value analysis

Robustness to unmeasured confounding was further evaluated using the E-value for the adjusted estimate and the lower bound of its 95% confidence interval (VanderWeele & Ding, 2017).

##### Sensitivity analyses

Sensitivity analyses were performed by excluding patients with extreme plasma S100A12 values (beyond the 1st and 99th percentiles).

We repeated the fully adjusted logistic regression (Model 3) using standardized S100A12 values (z-score). Odds ratios were interpreted per one standard deviation (SD) increase in S100A12, using the same covariates as in the main model.

##### Baseline risk–matched analysis

To further reduce baseline imbalance between patients who developed SA-AKI and those who did not, we performed 1:1 nearest-neighbor matching (caliper = 0.2) based on a multivariable baseline risk score constructed from age, gender, BMI, hypertension, coagulation disorder, SOFA score, CRP, and NT-proBNP.

After matching, covariate balance was assessed using standardized mean differences (SMDs). Because matching generated paired observations, conditional logistic regression was applied in the matched cohort. Variables with residual imbalance (SMD > 0.1) were further adjusted to assess the robustness of the association between plasma S100A12 and SA-AKI.

#### Sample size justification

A post-hoc power analysis was conducted using PASS 2021 (Logistic Regression, Wald test) based on the observed data from this study. The incidence of AKI was 59.9%, with a significance level of α = 0.05 and a moderate correlation of R^2^ = 0.15 between plasma S100A12 and other covariates. Power was estimated for a standardized effect size expressed as the odds ratio (OR) per one standard deviation (SD) increase in S100A12 concentration.

The standardized (per-SD) effect size was selected because it is scale-independent and aligns with the assumptions of the PASS logistic regression model.

## Results

### Patient selection and baseline characteristics

**Fig. 1:**
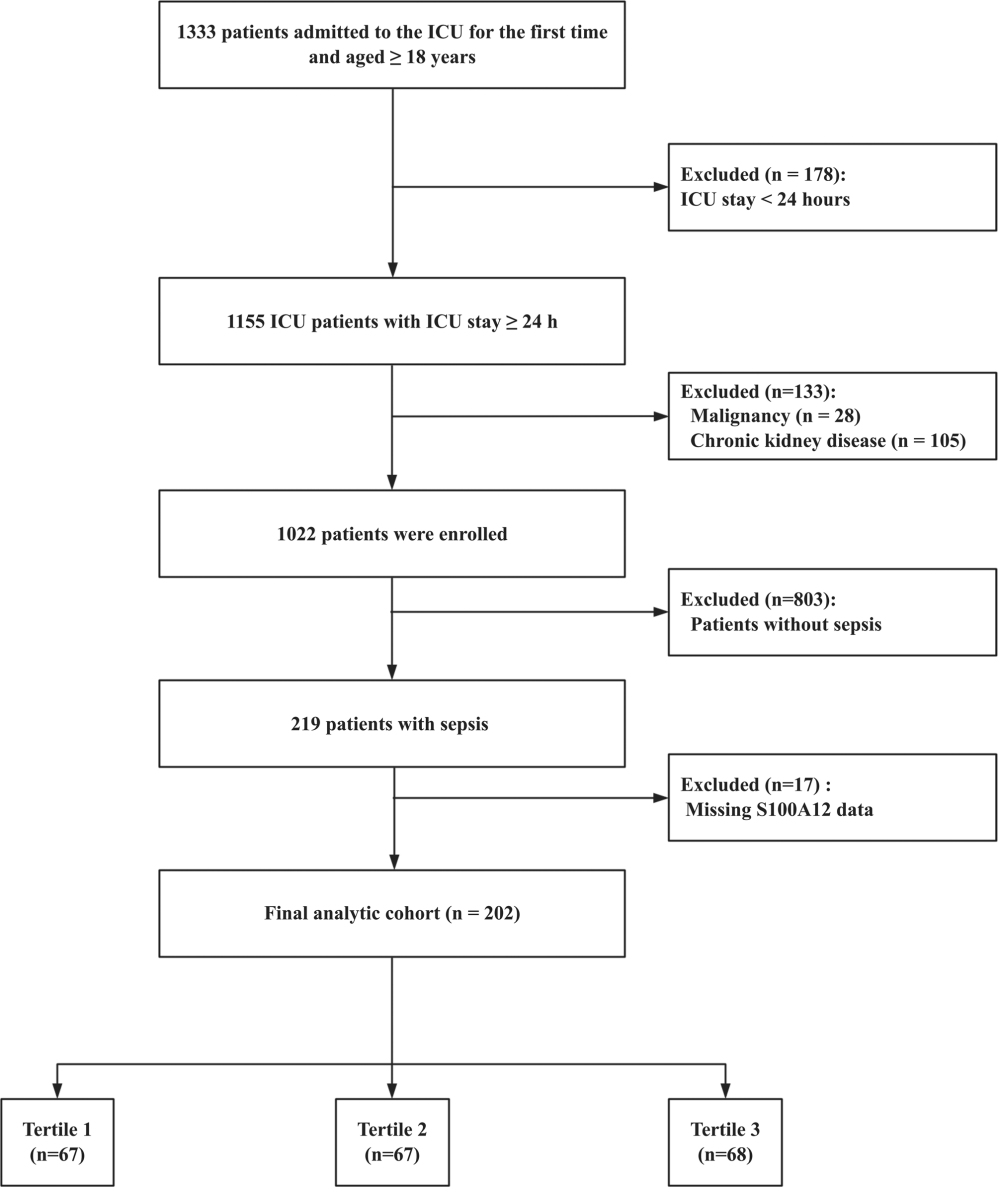
Flow diagram of patient enrollment and exclusion. A total of 1333 adult patients admitted to the ICU between September 2023 and June 2025 were screened for eligibility. Exclusion criteria included ICU stay < 24 hours (*n* = 178), pre-existing malignancy (*n* = 28), chronic kidney disease (*n* = 105), absence of sepsis diagnosis (*n* = 803), and missing plasma S100A12 measurements (*n* = 17). After applying these criteria, 202 patients with sepsis were enrolled and analyzed. Patients were further categorized into tertiles according to baseline S100A12 levels. Abbreviations: ICU, intensive care unit

After applying the predefined inclusion and exclusion criteria (Fig. 1), a total of 202 patients with sepsis were included from 1333 ICU admissions between September 2023 and June 2025. Patients were subsequently stratified into tertiles (T1–T3) based on baseline plasma S100A12 levels for comparative analysis.

Baseline characteristics are summarized in Table 1. The cohort consisted mainly of older adults (mean 67.8 ± 15.9 years), 58.4% of whom were male. With increasing S100A12 levels, higher SOFA and APACHE II scores, elevated PCT and creatinine levels, and more frequent vasopressor use were observed (all *p* < 0.05).

**Table 1:** Baseline characteristics of patients stratified by S100A12 tertiles

Variables	Overall (N = 202)	T1 (n = 67)	T2 (n = 67)	T3 (n = 68)	p value
Age, year, Mean ± SD	67.8 ± 15.9	67.3 ± 17.7	67.7 ± 14.8	68.3 ± 15.3	0.926
Gender, n (%)					0.742
Female	84 (41.6)	30 (44.8)	28 (41.8)	26 (38.2)	
Male	118 (58.4)	37 (55.2)	39 (58.2)	42 (61.8)	
BMI, kg/m^2^, Mean ± SD	23.0 ± 4.1	22.1 ± 3.3	23.7 ± 4.6	23.2 ± 4.3	0.062
DM, n (%)	58 (28.7)	16 (23.9)	20 (29.9)	22 (32.4)	0.536
HBP, n (%)	86 (42.6)	15 (22.4)	32 (47.8)	39 (57.4)	< 0.001
CVD, n (%)	69 (34.2)	25 (37.3)	18 (26.9)	26 (38.2)	0.304
SOFA, Mean ± SD	7.4 ± 3.7	6.0 ± 3.1	7.4 ± 3.6	8.6 ± 3.8	< 0.001
APACHE II, Mean ± SD	20.1 ± 6.2	18.6 ± 6.5	20.2 ± 5.4	21.6 ± 6.5	0.02
CRP, mg/L, Mean ± SD	150.7 ± 92.5	134.6 ± 87.9	159.4 ± 96.2	158.0 ± 92.5	0.22
Neutrophil count, ×10^9^/L, Mean ± SD	14.2 ± 7.7	14.2 ± 8.1	13.9 ± 7.1	14.5 ± 8.1	0.929
PCT, ng/mL, Median (IQR)	15.7 (3.2–52.6)	7.3 (0.7–27.5)	17.6 (4.7–51.7)	24.2 (8.0–86.3)	0.001
Cr, µmol/L, Median (IQR)	118.2 (68.9–195.2)	77.0 (54.0–99.7)	134.4 (70.0–194.5)	171.9 (119.0–255.6)	< 0.001
Coagulation disorder, n (%)	131 (64.9)	35 (52.2)	43 (64.2)	53 (77.9)	0.007
Albumin, g/L, Mean ± SD	27.9 ± 5.4	29.0 ± 5.4	27.2 ± 5.3	27.5 ± 5.2	0.122
NT-proBNP, pg/mL, Median (IQR)	3895 (1044–7009)	1950 (398–6856)	4659 (1882–7483)	5281 (1464– 12,607)	0.003
PaO₂, mmHg, Mean ± SD	91.5 ± 32.0	100.8 ± 36.5	86.3 ± 29.8	87.4 ± 27.5	0.013
Blood lactate, mmol/L, Median (IQR)	2.9 (1.8–4.8)	2.3 (1.6–4.2)	2.5 (2.0–6.2)	3.5 (2.1–4.8)	0.115
Blood glucose, mmol/L, Mean ± SD	14.0 ± 5.5	13.0 ± 5.7	14.0 ± 5.2	14.9 ± 5.5	0.118
Pulmonary infection, n (%)	50 (24.8)	16 (23.9)	17 (25.4)	17 (25.0)	0.979
Abdominal infection, n (%)	98 (48.5)	31 (46.3)	32 (47.8)	35 (51.5)	0.823
24-h urine output, mL, Median (IQR)	1750 (1100–2500)	1850 (1225–2580)	1604 (1048–2400)	1685 (1088–2475)	0.437
7-day fluid balance,mL, Median (IQR)	1896 (24–4183)	1830 (210–3668)	1942 (345–4383)	1855 (−930–4111)	0.551
CRRT, n (%)	37 (18.3)	6 (9.0)	17 (25.4)	14 (20.6)	0.041
MV, n (%)	102 (50.5)	27 (40.3)	35 (52.2)	40 (58.8)	0.093
Vasopressor therapy, n (%)	153 (75.7)	40 (59.7)	53 (79.1)	60 (88.2)	< 0.001
Nephrotoxic antibiotic exposure before AKI, n (%)	111 (55.0)	32 (47.8)	36 (53.7)	43 (63.2)	0.19
MAP, mmHg, Mean ± SD	68.0 ± 11.7	69.9 ± 10.9	68.6 ± 10.7	65.4 ± 13.1	0.065
ARDS, n (%)	75 (37.1)	20 (29.9)	26 (38.8)	29 (42.6)	0.288
AKI, n (%)	121 (59.9)	16 (23.9)	43 (64.2)	62 (91.2)	< 0.001
Septic shock, n (%)	150 (74.3)	35 (52.2)	52 (77.6)	63 (92.6)	< 0.001
Length of stay, days, Median (IQR)	10 (5–16)	10 (6–14)	10 (5–16)	11 (6–16)	0.853
ICU stay, days, Median (IQR)	5 (3–11)	5 (2–8)	6 (3–11)	6 (3–12)	0.193
28-day mortality, n (%)	43 (21.3)	7 (10.4)	19 (28.4)	17 (25.0)	0.027
Overall survival, days, Mean ± SD	23.4 ± 9.7	25.6 ± 7.4	21.9 ± 10.7	22.7 ± 10.4	0.07

Tertiles of plasma S100A12 were defined as follows: T1 ≤38.46 ng/mL; T2 38.47–52.20 ng/mL; T3 >52.20 ng/mL. Data are expressed as mean ± standard deviation (SD), median (interquartile range [IQR]), or number (percentage). Comparisons across S100A12 tertiles (T1–T3) were conducted using one-way ANOVA for normally distributed variables, the Kruskal–Wallis H test for non-normal variables, and χ² or Fisher’s exact test for categorical variables.Abbreviations: APACHE II, Acute Physiology and Chronic Health Evaluation II; ARDS, acute respiratory distress syndrome; BMI, body mass index; CVD, cardiovascular disease; CRP, C-reactive protein; Cr, serum creatinine; CRRT, continuous renal replacement therapy; DM, diabetes mellitus; HBP, hypertension; IQR, interquartile range; MAP, mean arterial pressure; MV, mechanical ventilation; NT-proBNP, N-terminal pro-brain natriuretic peptide; PaO₂, arterial oxygen pressure; PCT, procalcitonin; SD, standard deviation; SOFA, Sequential Organ Failure Assessment; T1–T3, S100A12 tertiles; ICU, intensive care unit

Hypertension, coagulation disorder, and septic shock also increased across tertiles (*p* < 0.01). Other demographic and biochemical variables were comparable.

When stratified by kidney function (Table 2), 121 (59.9%) patients developed SA-AKI. Compared with non-AKI cases, SA-AKI patients showed higher inflammatory and cardiac biomarkers (PCT, NT-proBNP, lactate), lower PaO₂, more frequent vasopressor and CRRT use, and higher 28-day mortality (all *p* < 0.01). Plasma S100A12 was significantly higher in the SA-AKI group (50.2 ± 8.0 vs 38.8 ± 6.8 ng/mL, *p* < 0.001).

**Table 2:** Baseline characteristics and clinical outcomes of patients with and without SA-AKI

Variables	Overall (N = 202)	Non-AKI (n = 81)	SA-AKI (n = 121)	p
Age, year,Mean ± SD	67.8 ± 15.9	68.8 ± 17.0	67.1 ± 15.2	0.451
Gender, n (%)				0.121
Female	84 (41.6)	39 (48.1)	45 (37.2)	
Male	118 (58.4)	42 (51.9)	76 (62.8)	
BMI, kg/m^2^, Mean ± SD	23.0 ± 4.1	22.2 ± 3.8	23.5 ± 4.3	0.023
DM, n (%)	58 (28.7)	23 (28.4)	35 (28.9)	0.935
HBP, n (%)	86 (42.6)	27 (33.3)	59 (48.8)	0.03
CVD, n (%)	69 (34.2)	28 (34.6)	41 (33.9)	0.92
SOFA, Mean ± SD	7.4 ± 3.7	5.7 ± 2.8	8.5 ± 3.8	< 0.001
APACH II, Mean ± SD	20.1 ± 6.2	18.2 ± 5.8	21.4 ± 6.2	< 0.001
CRP, mg/L, Mean ± SD	150.7 ± 92.5	131.9 ± 89.0	163.3 ± 93.1	0.018
Neutrophil count, 10^9^/L, Mean ± SD	14.2 ± 7.7	12.8 ± 6.5	15.1 ± 8.4	0.04
PCT, ng/ml, Median (IQR)	15.7 (3.2, 52.6)	7.2 (0.8, 21.3)	24.2 (8.0, 91.8)	< 0.001
Cr, umol/L, Median (IQR)	118.2 (68.9, 195.2)	66.0 (47.0, 85.6)	169.3 (130.0, 251.5)	< 0.001
Coagulation disorder, n (%)	131 (64.9)	37 (45.7)	94 (77.7)	< 0.001
Albumin, g/L, Mean ± SD	27.9 ± 5.4	28.4 ± 5.4	27.6 ± 5.3	0.31
NT-proBNP, pg/ml, Median (IQR)	3895.0 (1044.0, 7009.3)	2038.4 (434.0, 6220.0)	5500.0 (1834.3, 11,382.0)	< 0.001
PaO₂, mmHg, Mean ± SD	91.5 ± 32.0	100.1 ± 37.9	85.7 ± 26.0	0.002
Blood lactate, mmol/L, Median (IQR)	2.9 (1.8, 4.8)	2.1 (1.5, 3.9)	3.5 (2.1, 5.6)	< 0.001
Blood glucose, mmol/L, Mean ± SD	14.0 ± 5.5	12.8 ± 4.7	14.7 ± 5.8	0.016
Pulmonary infection, n (%)	50 (24.8)	22 (27.2)	28 (23.1)	0.516
Abdominal infection, n (%)	98 (48.5)	38 (46.9)	60 (49.6)	0.709
24-h urine output, mL, Median (IQR)	1750.0 (1100.0, 2500.0)	1850.0 (1300.0, 2600.0)	1620.0 (1000.0, 2430.0)	0.092
7-day fluid balance,mL, Median (IQR)	1895.5 (23.8, 4183.2)	1790.0 (180.0, 3090.0)	1940.0 (0.0, 4405.0)	0.435
CRRT, n (%)	37 (18.3)	5 (6.2)	32 (26.4)	< 0.001
MV, n (%)	102 (50.5)	33 (40.7)	69 (57)	0.023
Vasopressor therapy, n (%)	153 (75.7)	52 (64.2)	101 (83.5)	0.002
Nephrotoxic antibiotic exposure before AKI, n (%)	111 (55.0)	40 (49.4)	71 (58.7)	0.193
MAP, mmHg，Mean ± SD	68.0 ± 11.7	70.3 ± 10.5	66.4 ± 12.3	0.019
ARDS, n (%)	75 (37.1)	26 (32.1)	49 (40.5)	0.226
Septic shock, n (%)	150 (74.3)	47 (58)	103 (85.1)	< 0.001
Length of stay, days, Median (IQR)	10.0 (5.0, 16.0)	9.0 (5.0, 15.0)	11.0 (6.0, 17.0)	0.398
ICU stay, days, Median (IQR)	5.0 (3.0, 11.0)	5.0 (2.0, 8.0)	6.0 (3.0, 12.0)	0.035
28-day mortality, n (%)	43 (21.3)	11 (13.6)	32 (26.4)	0.029
Overall survival, days, Mean ± SD	23.4 ± 9.7	25.0 ± 7.9	22.3 ± 10.7	0.051
S100A12, ng/mL, Mean ± SD	45.6 ± 9.4	38.8 ± 6.8	50.2 ± 8.0	< 0.001

Continuous variables are expressed as mean ± standard deviation (SD) or median (interquartile range [IQR]); categorical variables are expressed as number (percentage). p values represent comparisons between non-AKI and SA-AKI groups using the independent-sample t test, Mann–Whitney U test, or χ² test, as appropriate.Abbreviations: APACHE II, Acute Physiology and Chronic Health Evaluation II; ARDS, acute respiratory distress syndrome; BMI, body mass index; CVD, cardiovascular disease; CRP, C-reactive protein; Cr, serum creatinine; CRRT, continuous renal replacement therapy; DM, diabetes mellitus; HBP, hypertension; IQR, interquartile range; MAP, mean arterial pressure; MV, mechanical ventilation; NT-proBNP, N-terminal pro-brain natriuretic peptide; PaO₂, arterial oxygen pressure; PCT, procalcitonin; S100A12, S100 calcium-binding protein A12; SA-AKI, sepsis-associated acute kidney injury; SD, standard deviation; SOFA, Sequential Organ Failure Assessment; ICU, intensive care unit

### Plasma S100A12 levels in sepsis and clinical subgroups

**Fig. 2:**
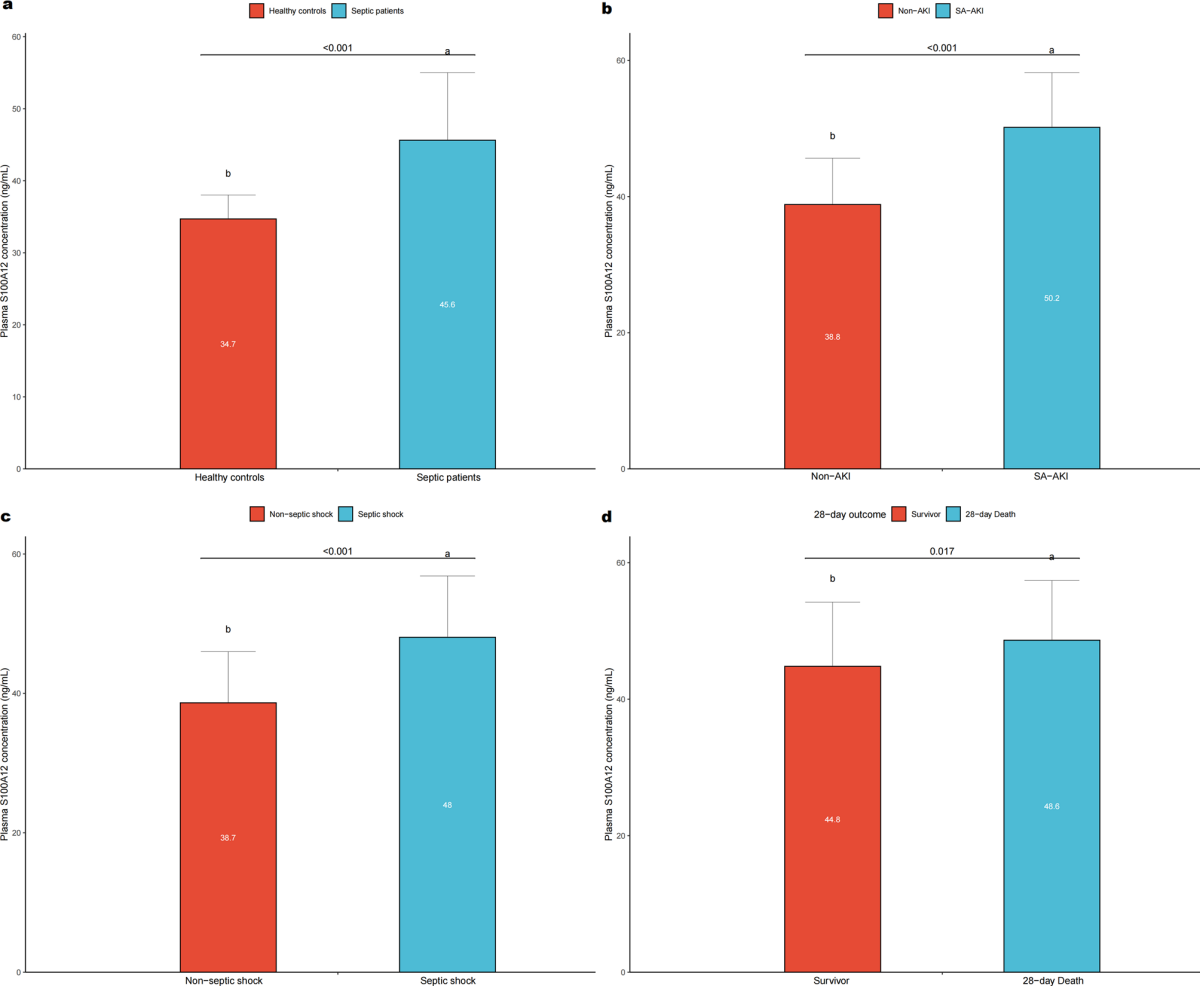
Plasma S100A12 concentrations in different clinical subgroups of patients with sepsis. Bar plots show plasma S100A12 concentrations measured within 24 hours after ICU admission in septic patients and various clinical subgroups. (**a**) plasma S100A12 levels were significantly higher in septic patients than in healthy controls (*p* < 0.001). (**b**) patients who developed SA-AKI had significantly higher S100A12 concentrations than those without AKI (*p* < 0.001). (**c**) S100A12 levels were elevated in patients with septic shock compared with those without shock (*p* < 0.001). (**d**) patients who died within 28 days exhibited higher S100A12 concentrations than 28-day survivors (*p* = 0.017). Each bar represents the mean ± SD; numerical values within bars indicate group means. Abbreviations: AKI, acute kidney injury; SA-AKI, sepsis-associated acute kidney injury; ICU, intensive care unit; SD, standard deviation

As illustrated in Fig. 2a–d, plasma S100A12 concentrations were significantly higher in septic patients than in healthy controls (*p* < 0.001; Fig. 2a). Among septic patients, S100A12 levels were markedly elevated in those who developed SA-AKI compared with those without AKI (*p* < 0.001; Fig. 2b). Similarly, patients with septic shock exhibited higher S100A12 concentrations than non-shock patients (*p* < 0.001; Fig. 2c).

In addition, non-survivors within 28 days had significantly higher S100A12 levels than survivors (*p* = 0.017; Fig. 2d).

### Univariate and multivariable logistic regression analyses (revised for results Section)

Univariate logistic regression (Table 3) identified several clinical and biochemical variables associated with SA-AKI. Among all factors, plasma S100A12 had the strongest association (OR = 1.18, 95% CI 1.13–1.23, *p* < 0.001).

**Table 3:** Univariate logistic regression analysis of factors associated with SA-AKI

Variable	OR (95% CI)	*p* **value**
Age	0.99 (0.98–1.01)	0.449
Gender: Male	1.57 (0.89–2.78)	0.122
BMI	1.09 (1.01–1.17)	0.025
DM	1.03 (0.55–1.91)	0.935
HBP	1.90 (1.06–3.41)	0.031
CVD	0.97 (0.54–1.75)	0.92
SOFA	1.31 (1.18–1.46)	< 0.001
APACHE II	1.10 (1.04–1.16)	< 0.001
CRP	1.00 (1.00–1.01)	0.019
Neutrophil count	1.04 (1.00–1.08)	0.042
PCT	1.02 (1.01–1.03)	< 0.001
Coagulation disorder	4.14 (2.25–7.63)	< 0.001
Albumin	0.97 (0.92–1.03)	0.309
NT-proBNP	1.00 (1.00–1.00)	0.001
PaO₂	0.99 (0.98–0.99)	0.003
Lactate	1.14 (1.03–1.27)	0.013
Blood glucose	1.07 (1.01–1.13)	0.018
Pulmonary infection	0.81 (0.42–1.54)	0.517
Abdominal infection	1.11 (0.63–1.96)	0.71
24-h urine output	1.00 (1.00–1.00)	0.109
7-day fluid balance	1.00 (1.00–1.00)	0.705
CRRT	1.93 (1.09–3.42)	0.024
Vasopressor therapy	2.82 (1.45–5.45)	0.002
Nephrotoxic antibiotic exposure before AKI	1.46 (0.83–2.56)	0.194
MAP	0.97 (0.95–1.00)	0.021
ARDS	1.44 (0.80–2.60)	0.227
Septic shock	4.14 (2.12–8.07)	< 0.001
**S100A12**	**1.18 (1.13–1.23)**	< 0.001

Each variable was tested individually to evaluate its association with SA-AKI. Data are expressed as odds ratios (ORs) with 95 % confidence intervals (CIs). Boldface indicates statistical significance (p < 0.05). Abbreviations:APACHE II, Acute Physiology and Chronic Health Evaluation II; ARDS, acute respiratory distress syndrome; BMI, body mass index; CVD, cardiovascular disease; CRP, C-reactive protein; CRRT, continuous renal replacement therapy; DM, diabetes mellitus; HBP, hypertension; MAP, mean arterial pressure; NT-proBNP, N-terminal pro-brain natriuretic peptide; PaO₂, arterial oxygen pressure; PCT, procalcitonin; SA-AKI, sepsis-associated acute kidney injury; SOFA, Sequential Organ Failure Assessment; S100A12, S100 calcium-binding protein A12

In multivariable models (Table 4), S100A12 remained independently associated with SA-AKI after sequential adjustment. Each 1 ng/mL increase in S100A12 corresponded to a 17% higher adjusted risk (*p* < 0.001). Patients in the second and third tertiles showed progressively higher odds compared with the lowest tertile (p for trend < 0.001).

**Table 4:** Multivariable logistic regression models evaluating the association between S100A12 and SA-AKI

**Variable**	**n.event (%)**	**Model 1**		**Model 2**		**Model 3 **	
		**OR (95 % CI) ** ***p*** ** value**		**OR (95 % CI) ** ***p*** ** value**		**OR (95 % CI) ** ***p*** ** value**	
**S100A12 (ng/mL)**	121 (59.9)	**1.18 (1.13 – 1.24)**	< 0.001	**1.18 (1.11 – 1.24)**	< 0.001	**1.17 (1.11 – 1.24)**	< 0.001
**Tertiles of S100A12**							
T1 (low)	16 (23.9)	1 (Ref)		1 (Ref)		1 (Ref)	
T2 (middle)	43 (64.2)	5.54 (2.52 – 12.22)	< 0.001	4.59 (1.77 – 11.88)	0.002	4.21 (1.59 – 11.13)	0.004
T3 (high)	62 (91.2)	34.96 (12.05 – 101.42)	< 0.001	30.08 (8.78 – 102.99)	< 0.001	25.10 (6.92 – 91.02)	< 0.001
***P*** ** for trend**			< 0.001		< 0.001		< 0.001

Model 1: adjusted for age, gender, body mass index (BMI), and hypertension (HBP)

Model 2: adjusted for Model 1 plus Sequential Organ Failure Assessment (SOFA) score, Acute Physiology and Chronic Health Evaluation II (APACHE II) score, C-reactive protein (CRP), neutrophil count, procalcitonin (PCT), coagulation disorder, N-terminal pro-brain natriuretic peptide (NT-proBNP), arterial oxygen pressure (PaO₂), blood lactate, and blood glucose

Model 3: adjusted for Model 2 plus mechanical ventilation (MV), vasopressor therapy, mean arterial pressure (MAP), septic shock, highly nephrotoxic antibiotic exposure before AKI, and 7-day cumulative fluid balance

Results are presented as odds ratios (ORs) with 95 % confidence intervals (CIs). Boldface indicates statistical significance (*p* < 0.05)

**Abbreviations: **APACHE II, Acute Physiology and Chronic Health Evaluation II; ARDS, acute respiratory distress syndrome; BMI, body mass index; CI, confidence interval; CRP, C-reactive protein; HBP, hypertension; MAP, mean arterial pressure; MV, mechanical ventilation; NT-proBNP, N-terminal pro-brain natriuretic peptide; OR, odds ratio; PaO₂, arterial oxygen pressure; PCT, procalcitonin; Ref, reference; SA-AKI, sepsis-associated acute kidney injury; SOFA, Sequential Organ Failure Assessment; S100A12, S100 calcium-binding protein A12

### Dose–response relationship between S100A12 and SA-AKI

**Fig. 3:**
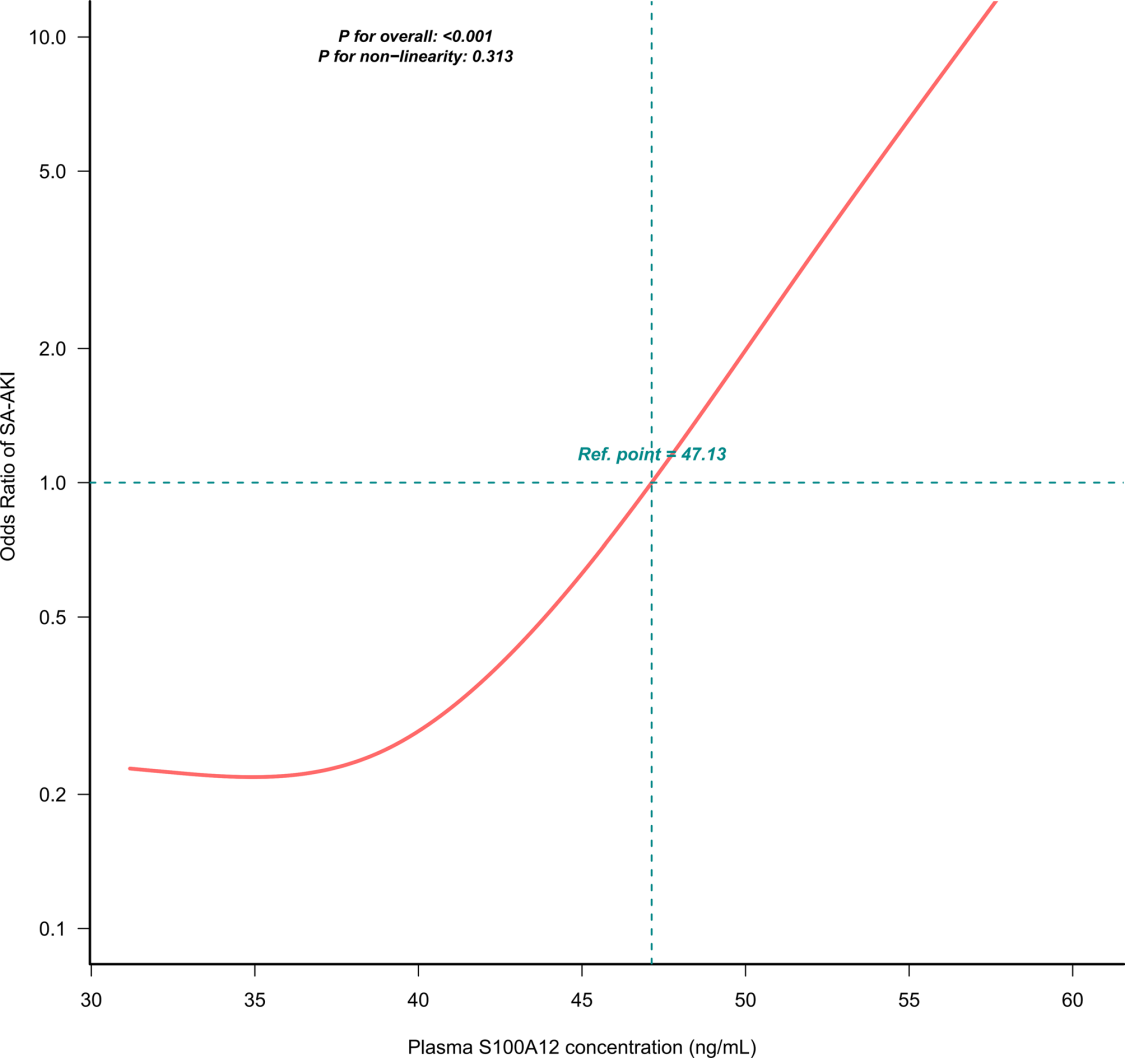
Dose-response association between plasma S100A12 levels and the risk of SA-AKI. RCS plot showing the adjusted association between plasma S100A12 concentration and the OR for SA-AKI. Multivariable logistic regression was adjusted for all covariates in Model 3, including demographic, severity, laboratory, and treatment variables. The red curve represents the estimated OR, and the dashed horizontal line indicates the reference level (OR = 1.0). Vertical dashed lines denote the reference point for S100A12 (47.13 ng/mL). There was a significant overall association (*p* < 0.001) and no evidence of non-linearity (*p* = 0.313). An increase in S100A12 was associated with a progressively higher risk of SA-AKI. Abbreviations: RCS, restricted cubic spline; OR, odds ratio; SA-AKI, sepsis-associated acute kidney injury

A restricted cubic spline analysis demonstrated a linear relationship between S100A12 and SA-AKI risk (p for overall < 0.001; p for non-linearity = 0.313; Fig. 3). The probability of SA-AKI increased steadily with higher S100A12 levels, with an inflection point around 47 ng/mL.

### Subgroup analysis

**Fig. 4:**
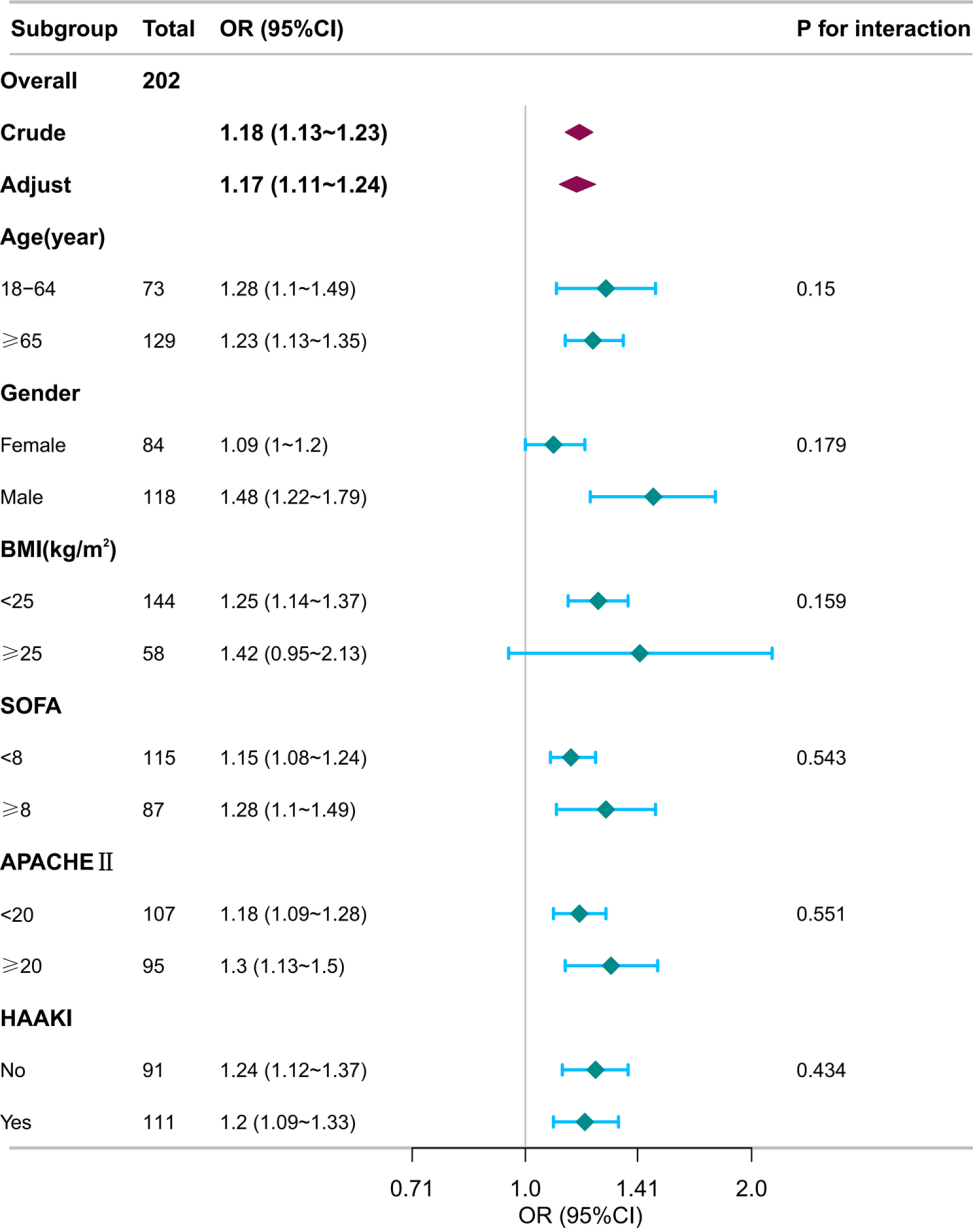
Subgroup analyses of the association between S100A12 levels and the risk of SA-AKI. Forest plot showing subgroup analyses of the association between plasma S100A12 concentration and sepsis-associated acute kidney injury (SA-AKI). Odds ratios (ORs) and 95% confidence intervals (CIs) were estimated from multivariable logistic regression models adjusted for all covariates in Model 3. The vertical dashed line indicates the null value (OR = 1.0). The overall association between S100A12 and SA-AKI remained significant after adjustment (*p* < 0.001). No significant interactions were observed across subgroups (all P for interaction > 0.05). Abbreviations: OR, odds ratio; CI, confidence interval; SA-AKI, sepsis-associated acute kidney injury; SOFA, Sequential Organ Failure Assessment; APACHE II, acute Physiology and Chronic Health Evaluation II; HAAKI, highly nephrotoxic antibiotic use before AKI onset

Subgroup analyses confirmed that the association between plasma S100A12 levels and the occurrence of SA-AKI was consistent across prespecified strata, including age, gender, BMI, disease severity, and treatment categories (Fig. 4). No significant interactions were observed between S100A12 and any subgroup variable (all p for interaction > 0.05).

Similarly, in subgroup analyses stratified by abdominal infection status, S100A12 remained significantly associated with SA-AKI in both subgroups, and no interaction was detected (p for interaction = 0.219; Supplementary Table S3).

### Time-to-event analysis

In the Cox proportional hazards analysis (Supplementary Table S4), higher plasma S100A12 levels were consistently associated with an increased hazard of SA-AKI. In the fully adjusted model (Model 3), each 1 ng/mL increase in S100A12 was associated with a 10% higher risk of SA-AKI (HR = 1.10, 95% CI 1.07–1.14; *p* < 0.001). When categorized into tertiles, the adjusted hazard ratios for the middle and highest tertiles were 2.72 (95% CI 1.46–5.08) and 8.09 (95% CI 4.17–15.69), respectively, with a significant linear trend (p for trend < 0.001).

**Fig. 5:**
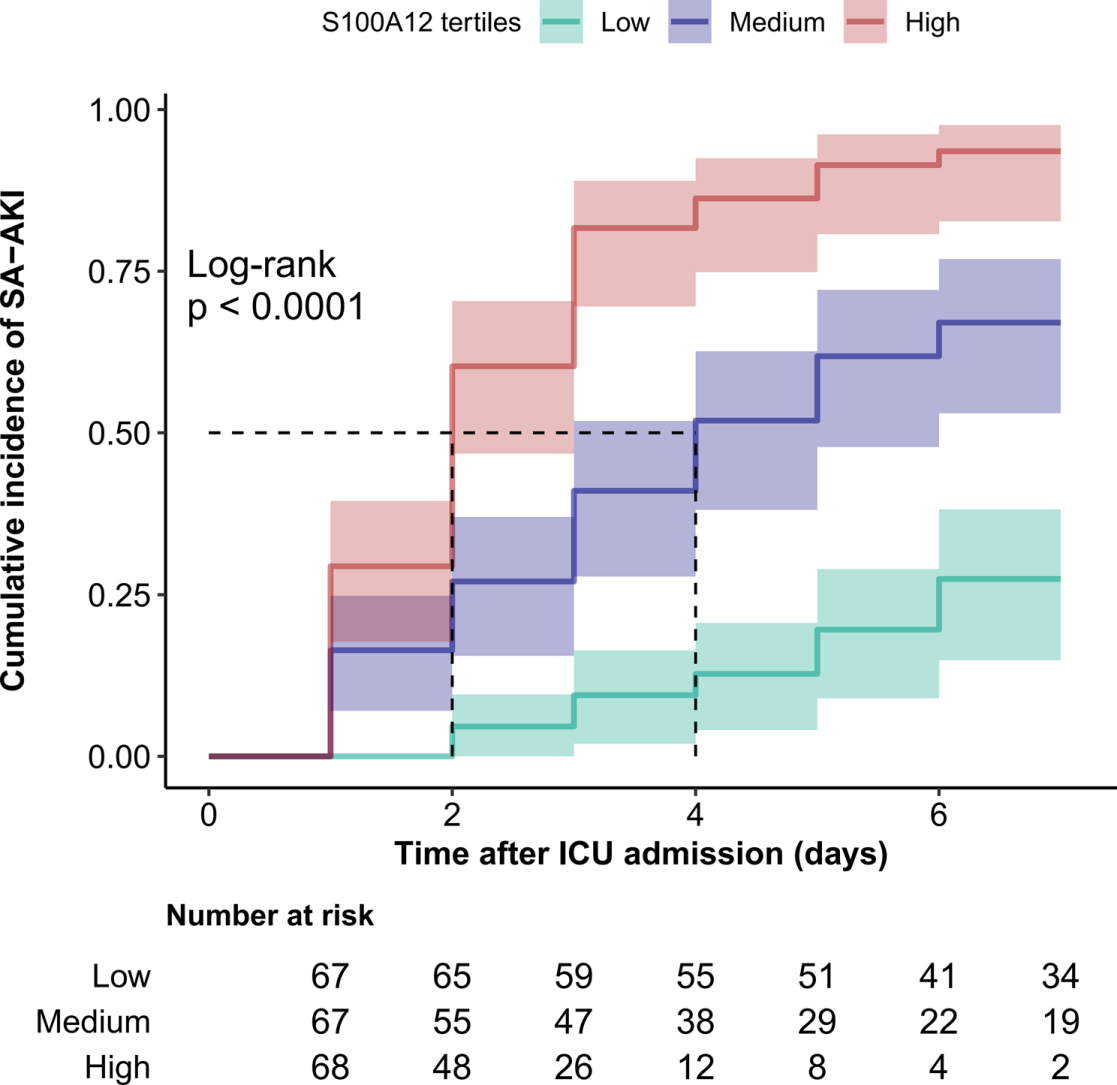
Kaplan–Meier curves for cumulative incidence of SA-AKI according to plasma S100A12 tertiles. Patients were stratified into tertiles based on baseline plasma S100A12 concentrations (low, medium, and high). Kaplan–Meier analysis showed a graded increase in the cumulative incidence of SA-AKI across tertiles, with the highest tertile (red line) exhibiting the earliest onset and greatest cumulative risk (log-rank *p* < 0.0001). Shaded areas represent 95% confidence intervals, and the table below indicates the number of patients remaining at risk at each time point. abbreviation: SA-AKI, sepsis-associated acute kidney injury

Kaplan–Meier analyses were conducted according to S100A12 tertiles (Fig. 5). Patients in the highest tertile exhibited the earliest onset and greatest cumulative incidence of SA-AKI, whereas those in the lowest tertile had the lowest risk. The cumulative incidence of SA-AKI increased progressively across tertiles, and the difference among groups was statistically significant (Log-rank *p* < 0.0001).

### ROC curve analysis

**Fig. 6:**
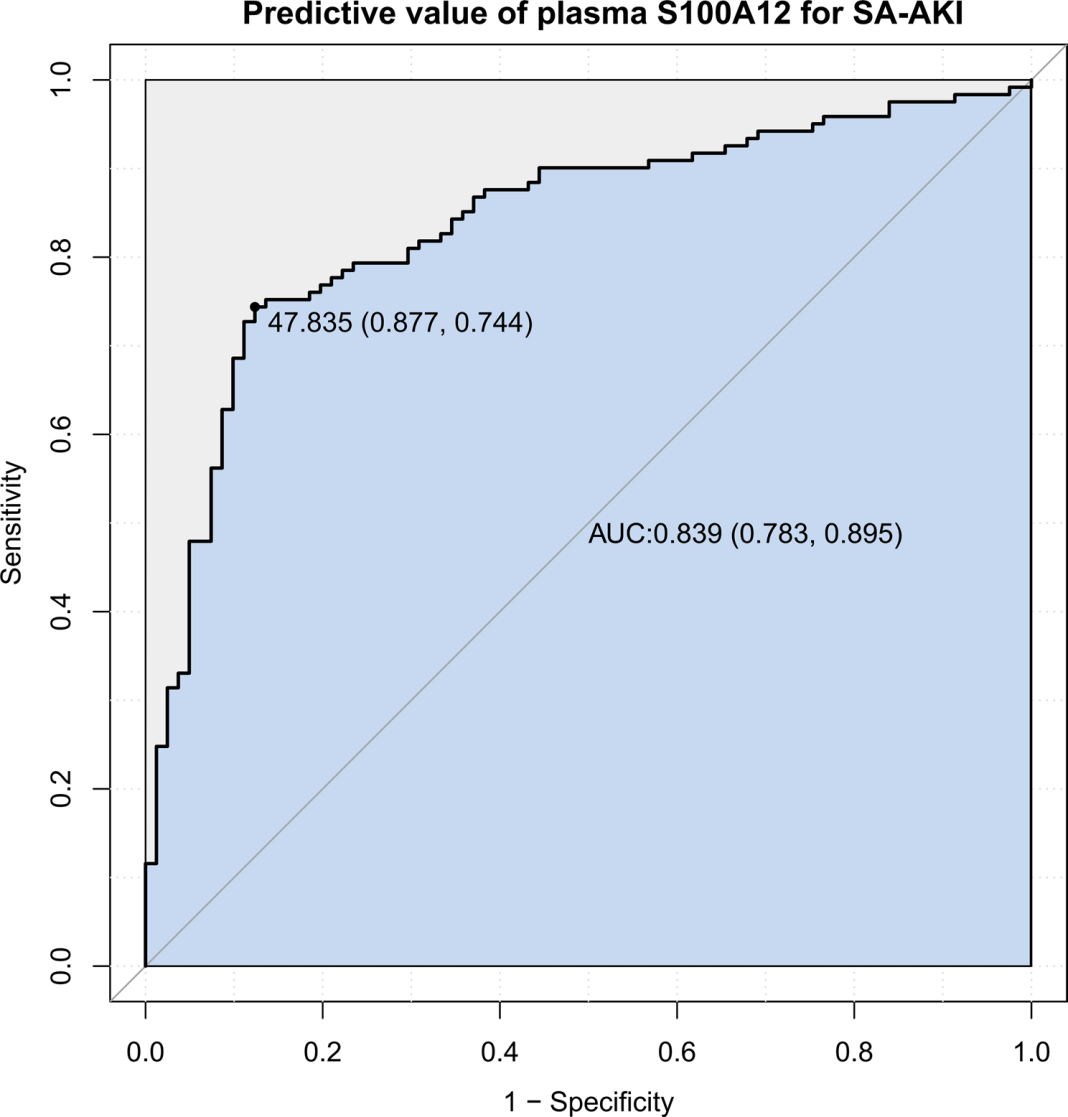
Predictive performance of plasma S100A12 for sepsis-associated acute kidney injury. Receiver operating characteristic (ROC) curve evaluating the predictive ability of plasma S100A12 measured within 24 hours after ICU admission for the development of sepsis-associated acute kidney injury (SA-AKI). The area under the curve (AUC) was 0.839 (95% CI 0.783–0.895), indicating good discriminative performance. The optimal cutoff value of 47.83 ng/mL (maximum Youden index = 0.621) corresponded to a sensitivity of 0.877 and a specificity of 0.744. The diagonal line represents the line of no discrimination (AUC = 0.5). The shaded area denotes the discriminative region under the curve. Abbreviations: AUC, area under the curve; CI, confidence interval; SA-AKI, sepsis-associated acute kidney injury; ICU, intensive care unit

ROC analysis showed that plasma S100A12 effectively discriminated SA-AKI (Fig. 6). The AUC was 0.839 (95% CI 0.783–0.895). The optimal cutoff (Youden index) was 47.83 ng/mL, with sensitivity 0.877 and specificity 0.744.

### Sensitivity analyses

After inclusion of variables reflecting medication exposure (HAAKI), nutritional status (BMI and ALB), and baseline renal function (Cr) into Model 4, S100A12 remained independently associated with SA-AKI (adjusted OR 1.16 [1.09–1.23], *p* < 0.001). Compared with the lowest tertile, the second and third tertiles showed progressively higher risks (adjusted OR 3.51 [1.23–9.98] and 24.0 [5.92–97.3]; p for trend < 0.001).

The E-value for the adjusted association was 1.38 (lower-CI E-value 1.29), indicating that an unmeasured confounder would need to have at least this strength of association with both S100A12 and SA-AKI to fully account for the observed effect. These results confirm the robustness of the main findings (Supplementary Table S1, S2).

After excluding patients with extreme plasma S100A12 values beyond the 1st and 99th percentiles, 196 patients were included in the re-analysis. The associations between S100A12 and SA-AKI remained statistically significant and consistent with the primary results across all adjustment models (Supplementary Table S5). In the fully adjusted model (Model 3), each 1 ng/mL increase in S100A12 was associated with an 18% higher risk of SA-AKI (adjusted OR = 1.18, 95% CI 1.11–1.25, *p* < 0.001), and the trend across tertiles remained significant (p for trend < 0.001).

In the sensitivity analysis using standardized S100A12 (per 1 SD increase), each one-standard-deviation increase in S100A12 was associated with a 4.62-fold higher unadjusted odds of SA-AKI (95% CI 3.08–6.91; *p* < 0.001) and a 4.38-fold higher adjusted odds after controlling for all Model 3 covariates (95% CI 2.55–7.52; *p* < 0.001) (Table S6).

After 1:1 nearest-neighbor matching based on baseline characteristics, 48 matched pairs (*n* = 96) were retained for analysis. Baseline characteristics were well balanced between the SA-AKI and non-SA-AKI groups after matching, with most SMDs below 0.1 (Supplementary Figure S1). Because matching generated paired observations, conditional logistic regression was performed in the matched cohort. Plasma S100A12 levels remained significantly associated with SA-AKI (OR = 1.18, 95% CI 1.07–1.29, *p* = 0.001). After further adjustment for variables with residual imbalance after matching (age, SOFA score, and NT-proBNP), the association remained robust (OR = 1.19, 95% CI 1.06–1.33, *p* = 0.002) (Supplementary Table S7).

### Sample size justification

Given an AKI incidence of 59.9%, a significance level of α = 0.05, and a moderate correlation (R^2^ = 0.15) among predictors, an odds ratio of 4.38 per one standard deviation (SD) increase in S100A12 corresponded to an estimated statistical power of approximately 90%.

## Discussion

This prospective ICU cohort study demonstrates that plasma S100A12 measured at ICU admission is elevated in patients with sepsis and further increased among those who subsequently develop SA-AKI. After adjustment for a broad range of clinical covariates, S100A12 remained independently associated with SA-AKI when analyzed as both a continuous and categorical variable, with generally consistent associations across predefined subgroups. Moreover, S100A12 showed good discriminatory performance for SA-AKI (AUC 0.839, 95% CI 0.783–0.895), supporting its potential role in early risk stratification in critically ill patients with sepsis.

Sepsis is a leading cause of AKI in the ICU, and renal dysfunction frequently develops early during the course of septic shock [23–25]. The pathogenesis of SA-AKI is complex and multifactorial, involving dysregulated systemic and renal inflammation, microcirculatory disturbances, endothelial dysfunction, mitochondrial injury, and metabolic dysregulation [6, 26–28]. Current diagnostic frameworks, including the KDIGO and Sepsis-3 criteria, primarily identify established kidney injury and may fail to capture early pathophysiological changes, thereby limiting timely recognition. Although several biomarkers—such as neutrophil gelatinase-associated lipocalin and kidney injury molecule-1—have been investigated, their sensitivity, reproducibility, and specificity in sepsis remain inconsistent across studies [17, 29–31]. These limitations underscore the need for biomarkers that are both clinically informative and mechanistically linked to SA-AKI.

S100A12, a calcium-binding protein secreted by activated neutrophils and monocytes, acts as a DAMP that engages RAGE and TLR4 receptors, activating downstream nuclear factor kappa-light-chain-enhancer of activated B cells (NF-κB) and mitogen-activated protein kinase (MAPK) signaling pathways [32–34]. These cascades amplify cytokine release, endothelial activation, and oxidative stress, leading to microvascular dysfunction and tubular epithelial injury—central events in SA-AKI pathogenesis. In addition, S100A12 may trigger NLRP3 (NACHT, LRR and PYD domains-containing protein 3) inflammasome activation and metabolic reprogramming, fostering a sustained pro-inflammatory state and aggravating renal injury [35–38]. Together, these mechanisms provide biological plausibility for the observed association between S100A12 and renal injury in sepsis.

Our findings differ from a prior report [18] that observed no significant differences in plasma S100A12 between sepsis patients with and without AKI. Discrepancies across studies may relate to differences in patient severity, timing of sampling, AKI definitions, and laboratory methods. In particular, our cohort comprised critically ill ICU patients and focused on sampling at ICU admission, a window that may better capture the early inflammatory surge in sepsis. Differences in assay platforms and pre-analytical handling may further contribute to heterogeneity. Together, these considerations highlight the importance of standardized sampling and analytic protocols when evaluating inflammatory biomarkers in sepsis.

From a clinical perspective, admission S100A12 measurement may complement established clinical indices by facilitating early risk awareness and identification of patients at increased risk of SA-AKI who may benefit from intensified renal monitoring and heightened attention to kidney-protective supportive care [19, 39–43]. Importantly, given the observational nature of this study, S100A12 should not be interpreted as a trigger for specific therapeutic interventions. Rather, elevated S100A12 levels at ICU admission may support risk stratification and reinforce adherence to kidney-protective supportive care in accordance with established sepsis and acute kidney injury management guidelines, such as optimization of hemodynamic status and avoidance or minimization of nephrotoxic exposures. At present, no S100A12-targeted therapy can be recommended based on these findings. Accordingly, its incremental value beyond conventional clinical predictors and existing biomarkers should be confirmed in external cohorts. Future multicenter studies with larger and more diverse populations, together with longitudinal sampling, are warranted to validate performance, clarify optimal thresholds, and determine whether trajectories of S100A12 improve risk assessment.

In summary, elevated plasma S100A12 at ICU admission is independently associated with subsequent SA-AKI in critically ill patients with sepsis and shows favorable discriminatory performance. These findings support S100A12 as a candidate biomarker for early risk stratification and are consistent with the involvement of neutrophil-driven inflammatory pathways in sepsis-related renal injury. Although the primary multivariable model included multiple covariates, extensive sensitivity analyses—including alternative model specifications, E-value assessment, exclusion of extreme values, standardized exposure analyses, and matched analyses with conditional logistic regression—consistently demonstrated stable associations, supporting the robustness of the findings.

This study has several limitations that should be acknowledged. First, as a single-center observational study, the findings may be subject to selection bias and limited generalizability; the exclusion of patients with chronic kidney disease or malignancy may further restrict external applicability. Second, although extensive multivariable adjustment was performed, residual confounding due to unmeasured factors cannot be fully excluded. Third, the relatively modest sample size may have limited statistical power, particularly for detecting smaller effect sizes. Fourth, S100A12 is not kidney-specific and may be elevated in other infectious or inflammatory conditions, which could reduce its diagnostic specificity for SA-AKI.

Furthermore, the cohort comprised a heterogeneous population of patients with sepsis, rather than an exclusively septic shock population, which should be considered when interpreting the severity profile of the study population. In addition, AKI severity staging was not separately analyzed in relation to S100A12 levels, which limits assessment of potential dose–response relationships across AKI stages. Finally, S100A12 was measured at a single time point, precluding assessment of temporal dynamics and limiting causal inference.

Despite these limitations, this study has several strengths. The prospective design enabled systematic data collection, and the concordant findings across multiple analytical approaches support the robustness of the observed association. Future multicenter studies with larger cohorts and longitudinal sampling, together with mechanistic investigations, are needed to validate these findings and clarify the biological and clinical significance of S100A12 in sepsis-associated acute kidney injury.

## Conclusions

This prospective study demonstrated that elevated S100A12 levels at ICU admission were independently associated with the development of sepsis-associated acute kidney injury. These findings suggest that S100A12 may reflect inflammatory and endothelial perturbations involved in the pathophysiology of SA-AKI. However, given the observational design of the study, S100A12 should be interpreted as an associative biomarker rather than a definitive predictive or causal factor. Further multicenter studies with larger cohorts, as well as mechanistic investigations, are warranted to validate these findings and to better elucidate the biological role of S100A12 in sepsis-related renal injury.

## Electronic supplementary material

Below is the link to the electronic supplementary material.

Supplementary Material 1

Supplementary Material 2

## Data Availability

The datasets generated and/or analyzed during the current study are available from the corresponding author upon reasonable request.

## Abbreviations

AKI: Acute kidney injury
ANOVA: Analysis of variance
APACHE II: Acute Physiology and Chronic Health Evaluation II
ARDS: Acute respiratory distress syndrome
AUC: Area under the curve
BMI: Body mass index
CI: Confidence interval
CKD: Chronic kidney disease
CRP: C-reactive protein
Cr: Serum creatinine
CRRT: Continuous renal replacement therapy
CVD: Cardiovascular disease
DAMP: Damage-associated molecular pattern
DM: Diabetes mellitus
ELISA: Enzyme-linked immunosorbent assay
HAAKI: Highly nephrotoxic antibiotic exposure before AKI
HBP: Hypertension
ICU: Intensive care unit
IQR: Interquartile range
KDIGO: Kidney Disease: Improving Global Outcomes
MAP: Mean arterial pressure
MAPK: Mitogen-activated protein kinase
MV: Mechanical ventilation
NF-κB: Nuclear factor kappa-light-chain-enhancer of activated B cells
NLRP3: NOD-like receptor family pyrin domain-containing 3 (inflammasome)
NT-proBNP: N-terminal pro-brain natriuretic peptide
OR: odds ratio
PaO₂: Arterial oxygen partial pressure
PCT: Procalcitonin
PSM: Propensity score matching
RAGE: Receptor for advanced glycation end-products
RCS: Restricted cubic spline
ROC: Receiver operating characteristic
SA-AKI: Sepsis-associated acute kidney injury
SD: Standard deviation
SMD: Standardized mean difference
SOFA: Sequential Organ Failure Assessment
S100A12: S100 calcium-binding protein A12
STROBE: Strengthening the Reporting of Observational Studies in Epidemiology
TLR4: Toll-like receptor 4.
