# Supplementary figures and images for: Association between S100 calcium-binding protein A12 and sepsis associated-acute kidney injury: a prospective cohort study
Source: BMC Nephrol. 2026 Jan 22;27:118. doi: 10.1186/s12882-026-04760-0 (PMC12911131; doi:10.1186/s12882-026-04760-0)

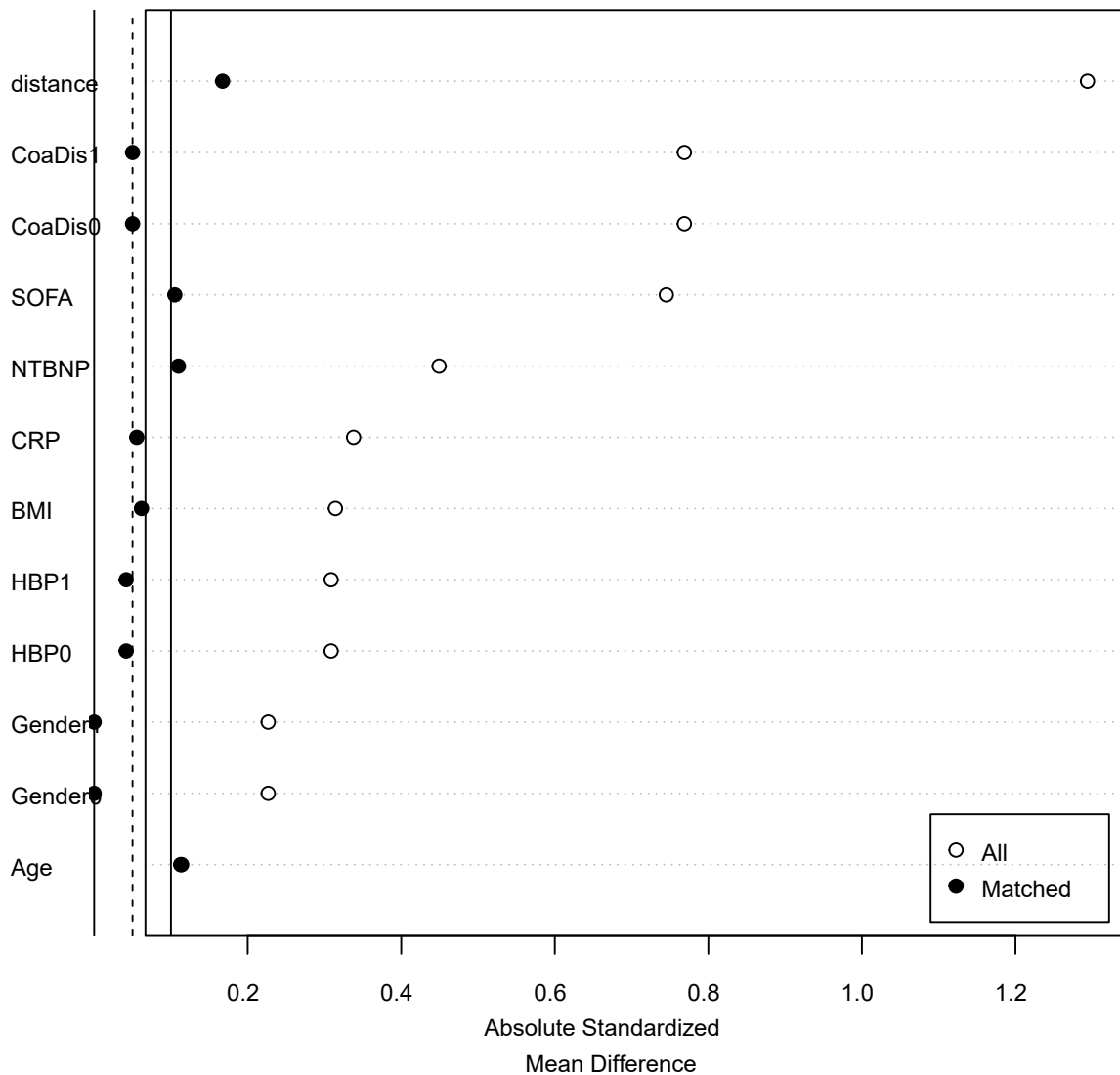

Supplement: Supplementary file 1 — Supplementary Material 1 [file 12882_2026_4760_MOESM1_ESM.pdf]
