# Supplementary material for: Association between S100 calcium-binding protein A12 and sepsis associated-acute kidney injury: a prospective cohort study
Source: BMC Nephrol. 2026 Jan 22;27:118. doi: 10.1186/s12882-026-04760-0 (PMC12911131; doi:10.1186/s12882-026-04760-0)
Supplement: Supplementary file 2 — Supplementary Material 2 [file 12882_2026_4760_MOESM2_ESM.docx]

**Table S1. E-value analysis for the robustness of the association between S100A12 and SA-AKI**

| **Item** | **point** | **lower** | **upper** |
| --- | --- | --- | --- |
| RR | 1.0816653826392 | 1.05356537528527 | 1.113552873 |
| E-values | 1.37887678444068 | 1.29112510401623 | NA |

**Table footnote**

The E-value quantifies the minimum strength of association that an unmeasured confounder would need to have with bothS100 calcium-binding protein A12 (S100A12) and sepsis-associated acute kidney injury (SA-AKI) to fully explain the observed association, conditional on measured covariates.

The adjusted relative risk (RR) of 1.08 corresponds to an E-value of 1.38 for the point estimate and 1.29 for the lower bound of its 95% confidence interval (CI).

Calculations followed the method of VanderWeele and Ding (Ann Intern Med, 2017).

**Abbreviations:** S100A12, S100 calcium-binding protein A12; RR, relative risk; CI, confidence interval; SA-AKI, sepsis-associated acute kidney injury.

**Table S2. Multivariable logistic regression evaluating the independent association between plasma S100A12 and SA-AKI.**

| **Variable** | **n.event_%** | **crude.OR (95%CI)p value** | **adj.OR (95%CI)p value** |
| --- | --- | --- | --- |
| **S100A12 (ng/mL)** | 121 (59.9) | 1.18 (1.13~1.23)  <0.001 | 1.16 (1.09~1.23)  <0.001 |
| **Tertiles of S100A12** |  |  |  |
| T1 (low) | 16 (23.9) | 1(Ref) | 1(Ref) |
| T2 (middle) | 43 (64.2) | 5.71 (2.69~12.11)  <0.001 | 3.51 (1.23~9.98)  0.019 |
| T3 (high) | 62 (91.2) | 32.94 (12.01~90.32)  <0.001 | 24 (5.92~97.3)  <0.001 |
| **P for trend** | 121 (59.9) | 5.73 (3.53~9.32)  <0.001 | 4.67 (2.38~9.15)  <0.001 |

**Table footnote**

The adjusted model (Model 4) was controlled for age, gender, body mass index (BMI), hypertension (HBP), Sequential Organ Failure Assessment (SOFA) score, C-reactive protein (CRP), lactate, mean arterial pressure (MAP), highly nephrotoxic antibiotic exposure before AKI (HAAKI), serum albumin, baseline creatinine (Cr), and 7-day cumulative fluid balance.

**Abbreviations:** S100A12, S100 calcium-binding protein A12; SA-AKI, sepsis-associated acute kidney injury; OR, odds ratio; CI, confidence interval; BMI, body mass index; HBP, hypertension; SOFA, Sequential Organ Failure Assessment; CRP, C-reactive protein; MAP, mean arterial pressure; HAAKI, highly nephrotoxic antibiotic exposure before AKI; Cr, creatinine.

**Table S3. Subgroup analysis by infection site (abdominal vs. non-abdominal)**

| **Subgroup** | **n.total** | **n.event_%** | **crude.OR_95CI** | **crude.P_value** | **adj.OR_95CI** | **adj.P_value** | **P.for.interaction** |
| --- | --- | --- | --- | --- | --- | --- | --- |
| AI |  |  |  |  |  |  |  |
| No | 105 | 62 (59) | 1.15 (1.09~1.22) | <0.001 | 1.2 (1.08~1.32) | <0.001 | 0.219 |
| Yes | 97 | 59 (60.8) | 1.21 (1.13~1.3) | <0.001 | 1.53 (1.19~1.98) | 0.001 |  |

**Table footnote**

Subgroup logistic regression of S100A12 for predicting sepsis-associated acute kidney injury (SA-AKI) by infection site. The interaction term between S100A12 and abdominal infection status (AI) tested effect modification.

**Abbreviations:** S100A12, S100 calcium-binding protein A12; SA-AKI, sepsis-associated acute kidney injury; OR, odds ratio; CI, confidence interval. AI, abdominal infection.

**Table S4. Association between plasma S100A12 and time to SA-AKI in the Cox proportional hazards model**

| **Variable** | **n.event_%** | **crude** | | **Model 1** | | **Model 2** | | **Model 3** | |
| --- | --- | --- | --- | --- | --- | --- | --- | --- | --- |
|  |  | **HR(95%CI)pvalue** | | **HR(95%CI) p value** | | **HR(95%CI)pvalue** | | **HR(95%CI)pvalue** | |
| **S100A12 (ng/mL)** | 121 (59.9) | 1.12 (1.09~1.15) | <0.001 | 1.12 (1.09~1.15) | <  0.001 | 1.1 (1.07~1.13) | <  0.001 | 1.1 (1.07~1.14) | <  0.001 |
| **Tertiles of S100A12** |  |  |  |  |  |  |  |  |  |
| T1 (low) | 16 (23.9) | 1(Ref) |  | 1(Ref) |  | 1(Ref) |  | 1(Ref) |  |
| T2 (middle) | 43 (64.2) | 3.84 (2.16~6.83) | <0.001 | 3.82 (2.13~6.85) | <  0.001 | 2.87 (1.57~5.23) | 0.001 | 2.72 (1.46~5.08) | 0.002 |
| T3 (high) | 62 (91.2) | 10.49 (5.96~18.47) | <0.001 | 10.64 (5.9~19.18) | <  0.001 | 7.69 (4.18~14.13) | <  0.001 | 8.09 (4.17~15.69) | <  0.001 |
| **P for trend** |  | 3.11 (2.41~4.01) | <0.001 | 3.14 (2.4~4.1) | <  0.001 | 2.75 (2.07~3.65) | <  0.001 | 2.88 (2.1~3.94) | <  0.001 |

**Table footnote**

Model 1: adjusted for age, gender, body mass index (BMI), and hypertension (HBP).

Model 2: adjusted for Model 1 plus Sequential Organ Failure Assessment (SOFA) score, Acute Physiology and Chronic Health Evaluation II (APACHE II) score, C-reactive protein (CRP), neutrophil count, procalcitonin (PCT), coagulation disorder, N-terminal pro-brain natriuretic peptide (NT-proBNP), arterial oxygen pressure (PaO₂), blood lactate, and blood glucose.

Model 3: adjusted for Model 2 plus mechanical ventilation (MV), vasopressor therapy, mean arterial pressure (MAP), septic shock, highly nephrotoxic antibiotic exposure before AKI, and 7-day cumulative fluid balance.

Results are presented as odds ratios (HRs) with 95 % confidence intervals (CIs). Boldface indicates statistical significance (p < 0.05).

**Abbreviations:** APACHE II, Acute Physiology and Chronic Health Evaluation II; ARDS, acute respiratory distress syndrome; BMI, body mass index; CI, confidence interval; CRP, C-reactive protein; HBP, hypertension; HR, hazard ratio; MAP, mean arterial pressure; MV, mechanical ventilation; NT-proBNP, N-terminal pro-brain natriuretic peptide; PaO₂, arterial oxygen pressure; PCT, procalcitonin; Ref, reference; SA-AKI, sepsis-associated acute kidney injury; SOFA, Sequential Organ Failure Assessment; S100A12, S100 calcium-binding protein A12.

**Table S5. Logistic regression analysis after excluding extreme plasma S100A12 values (1st and 99th percentiles)**

| **Variable** | **n.event_%** | **crude** | | **Model 1** | | **Model 2** | | **Model 3** | |
| --- | --- | --- | --- | --- | --- | --- | --- | --- | --- |
|  |  | **OR(95%CI) p value** | | **OR(95%CI) p value** | | **OR(95%CI)pvalue** | | **OR(95%CI)pvalue** | |
| **S100A12 (ng/mL)** | 117 (59.7) | 1.18 (1.13~1.23) | <0.001 | 1.19 (1.13~1.25) | <  0.001 | 1.18 (1.11~1.24) | <  0.001 | 1.18 (1.11~1.25) | <  0.001 |
| **Tertiles of S100A12** |  |  |  |  |  |  |  |  |  |
| T1 (low) | 16 (24.6) | 1(Ref) |  | 1(Ref) |  | 1(Ref) |  | 1(Ref) |  |
| T2 (middle) | 41 (63.1) | 5.23 (2.46~11.15) | <0.001 | 5.02 (2.27~11.08) | <  0.001 | 3.79 (1.46~9.82) | 0.006 | 3.65 (1.36~9.83) | 0.01 |
| T3 (high) | 60 (90.9) | 30.62 (11.14~84.18) | <0.001 | 32.58 (11.22~94.64) | <  0.001 | 27.14 (7.98~92.25) | <  0.001 | 24.22 (6.69~87.7) | <  0.001 |
| **P for trend** |  | 5.48 (3.37~8.89) | <0.001 | 5.59 (3.35~9.32) | <  0.001 | 5.02 (2.78~9.06) | <  0.001 | 4.75 (2.55~8.88) | <  0.001 |

**Table S6. Association between standardized plasma S100A12 (per 1 SD) and risk of SA-AKI**

| **Variable** | **N** | **n.event_%** | **Crude OR**  **(95%CI)** | **P value** | **Adjusted OR**  **(95%CI)** | **P value** |
| --- | --- | --- | --- | --- | --- | --- |
| S100A12 (per 1 SD, z-score) | 202 | 121 (59.9) | 4.62 (3.08~6.91) | <0.001 | 4.38 (2.55~7.52) | <0.001 |

**Table footnote**

Odds ratios (ORs) were derived from logistic regression analyses. The adjusted model corresponds to Model 3, which included the following covariates: age, gender, BMI, hypertension, SOFA, APACHE II, CRP, neutrophil count, PCT, coagulation disorder, NT-proBNP, PaO₂, lactate, glucose, mechanical ventilation, vasopressor therapy, MAP, septic shock, HAAKI, and 7-day cumulative fluid balance.

S100A12 was standardized to a z-score; odds ratios (ORs) are expressed per one standard deviation (SD) increase in plasma S100A12 concentration.

**Abbreviations:**

AKI, acute kidney injury; APACHE II, Acute Physiology and Chronic Health Evaluation II; BMI, body mass index; CRP, C-reactive protein; HAAKI, highly nephrotoxic antibiotic exposure before AKI; MAP, mean arterial pressure; NT-proBNP, N-terminal pro-brain natriuretic peptide; OR, odds ratio; PaO₂, arterial oxygen pressure; PCT, procalcitonin; SA-AKI, sepsis-associated acute kidney injury; SD, standard deviation; SOFA, Sequential Organ Failure Assessment.

**Table S7. Association between plasma S100A12 and SA-AKI in the matched cohort**

| **Variable** | **n.event_%** | **Matched model** | | **Adjusted matched model** | |
| --- | --- | --- | --- | --- | --- |
|  |  | **OR(95%CI) p value** | | **OR(95%CI) p value** | |
| **S100A12 (ng/mL)** | 48 (50) | 1.18 (1.07~1.29) | 0.001 | 1.19 (1.06~1.33) | 0.002 |
| **Tertiles of S100A12** |  |  |  |  |  |
| T1 (low) | 9 (25) | 1(Ref) |  | 1(Ref) |  |
| T2 (middle) | 20 (54.1) | 6.76 (1.46~31.23) | 0.014 | 9.98 (1.78~55.83) | 0.009 |
| T3 (high) | 19 (82.6) | 15.86 (3.07~81.95) | 0.001 | 23.41 (3.38~162.14) | 0.001 |
| **P for trend** |  | 3.76 (1.74~8.13) | 0.001 | 4.42 (1.81~10.79) | 0.001 |

**Table footnote**

Conditional logistic regression analyses were performed in the matched cohort (n = 96). Matching was conducted using a 1:1 nearest-neighbor algorithm with a caliper width of 0.2 to balance baseline characteristics between SA-AKI and non–SA-AKI patients. The matched model represents conditional logistic regression accounting for the matched design. The adjusted matched model further adjusted for covariates with residual imbalance after matching (age, SOFA score, and NT-proBNP).

P for trend was calculated across S100A12 tertiles.

**Abbreviations:** OR, odds ratio; CI, confidence interval; SOFA, Sequential Organ Failure Assessment; NT-proBNP, N-terminal pro–B-type natriuretic peptide.
